# Spatially-segmented undersampled MRI temperature reconstruction for transcranial MR-guided focused ultrasound

**DOI:** 10.1186/s40349-017-0092-0

**Published:** 2017-05-30

**Authors:** Pooja Gaur, Beat Werner, Xue Feng, Samuel W. Fielden, Craig H. Meyer, William A. Grissom

**Affiliations:** 1 Department of Radiology, Stanford University, Stanford, USA; 2 Center for MR-Research, University Children’s Hospital, Zurich, Switzerland; 3 Department of Biomedical Engineering, University of Virginia, Charlottesville, USA; 4 Autism and Developmental Medicine Institute, Geisinger Health System, Danville, USA; 5 Institute of Imaging Science, Vanderbilt University, 1161 21st Ave S, Nashville, 37232 USA; 6 Department of Biomedical Engineering, Vanderbilt University, 21st Ave S, Nashville, 37232 USA

**Keywords:** Temperature imaging, Image reconstruction, Proton resonance frequency-shift, Thermometry, MRI-guided focused ultrasound

## Abstract

**Background:**

Volumetric thermometry with fine spatiotemporal resolution is desirable to monitor MR-guided focused ultrasound (MRgFUS) procedures in the brain, but requires some form of accelerated imaging. Accelerated MR temperature imaging methods have been developed that undersample k-space and leverage signal correlations over time to suppress the resulting undersampling artifacts. However, in transcranial MRgFUS treatments, the water bath surrounding the skull creates signal variations that do not follow those correlations, leading to temperature errors in the brain due to signal aliasing.

**Methods:**

To eliminate temperature errors due to the water bath, a spatially-segmented iterative reconstruction method was developed. The method fits a k-space hybrid signal model to reconstruct temperature changes in the brain, and a conventional MR signal model in the water bath. It was evaluated using single-channel 2DFT Cartesian, golden angle radial, and spiral data from gel phantom heating, and in vivo 8-channel 2DFT data from a FUS thalamotomy. Water bath signal intensity in phantom heating images was scaled between 0-100% to investigate its effect on temperature error. Temperature reconstructions of retrospectively undersampled data were performed using the spatially-segmented method, and compared to conventional whole-image k-space hybrid (phantom) and SENSE (in vivo) reconstructions.

**Results:**

At 100% water bath signal intensity, 3 ×-undersampled spatially-segmented temperature reconstruction error was nearly 5-fold lower than the whole-image k-space hybrid method. Temperature root-mean square error in the hot spot was reduced on average by 27 × (2DFT), 5 × (radial), and 12 × (spiral) using the proposed method. It reduced in vivo error 2 × in the brain for all acceleration factors, and between 2 × and 3 × in the temperature hot spot for 2-4 × undersampling compared to SENSE.

**Conclusions:**

Separate reconstruction of brain and water bath signals enables accelerated MR temperature imaging during MRgFUS procedures with low errors due to undersampling using Cartesian and non-Cartesian trajectories. The spatially-segmented method benefits from multiple coils, and reconstructs temperature with lower error compared to measurements from SENSE-reconstructed images. The acceleration can be applied to increase volumetric coverage and spatiotemporal resolution.

## Background

Over the last ten years, MR-guided focused ultrasound (MRgFUS) has emerged as a promising treatment modality for several neurological conditions. Targeted thermal heating delivered by MRgFUS is being used to treat conditions such as essential tremor [1–3], chronic neuropathic pain [4], Parkinson’s disease [5], obsessive compulsive disorder [6], and brain tumors [7, 8]. In cases targeting subcortical areas near the center of the brain, the potential benefits of MRgFUS therapy are promising. With no incisions, the risk of damage to surrounding brain structures and cortical tissue is dramatically lower than with invasive procedures. For this reason, MRgFUS may be the only treatment option in otherwise inoperable situations [7, 9].

**Fig. 1:**
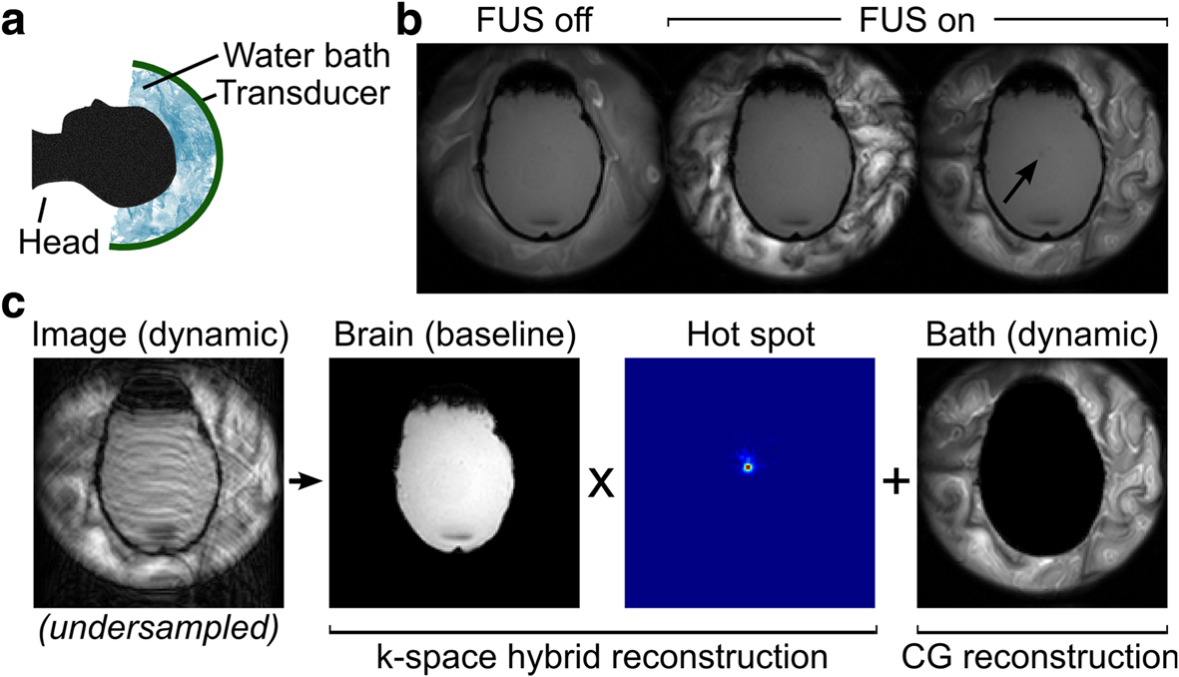
Overview of transcranial MRgFUS setup, treatment images, and reconstruction model. **a** During MRgFUS treatment, the *patient’s head* is immobilized in the transducer and circulating *water bath*. **b** The *water bath* signal exhibits random dynamic changes during sonication (*arrow* indicates sonication target in gel phantom). **c** The proposed spatially-segmented reconstruction model for undersampled brain MRgFUS, which separately estimates a *water bath image* without a *baseline*, and a temperature map in the brain with a *baseline*. In the proposed method, undersampled dynamic data are reconstructed using the *k-space hybrid* method in the brain and a conjugate gradient (CG) method in the *water bath*

Current clinical transcranial MRgFUS systems comprise a hemispheric 1024-element ultrasound phased array transducer with 30 cm diameter (Insightec ExAblate Neuro 4000; Insightec Ltd, Haifa, Israel). The patient’s head is positioned in the device and immobilized by a stereotactic frame. Degassed water fills the space between the transducer and the head, and is contained by a rubber membrane that allows direct contact between the water and scalp [9]. Figure 1 a illustrates a cross-sectional view of the transducer and water bath positioned around the patient’s head. The water bath couples ultrasound energy between the transducer and the body, and is chilled to 15-20 °C and circulated after each sonication to dissipate heat from the head. Although active water circulation is performed between imaging sequences, residual circulatory flow and acoustic streaming effects during sonication cause motion of the water bath during imaging. This intra-scan motion of the water bath results in artifacts with a ripple-like appearance that alias into the MR images and temperature maps.

The current clinical temperature monitoring protocol for transcranial MRgFUS dynamically images a single 2D slice. Increased spatial coverage is needed to enable monitoring of off-target heating and to evaluate new treatment targets [10, 11], but this will require some form of accelerated temperature imaging to acquire more data without compromising frame rate. Accelerated temperature imaging could also reduce temperature errors due to intra-scan water motion artifacts. However, conventional MRI scan acceleration approaches such as parallel imaging [12, 13] and simultaneous multi-slice imaging [14–16] require a dense array of receive coils to be placed near the head, so they are of limited utility in MRgFUS applications because coil placement is restricted by the transducer. As will be shown here, the sensitivity profiles of coils placed outside the transducer (far away from the head), are not sufficiently distinct in the brain to provide artifact-free images and temperature maps at useful scan acceleration factors using conventional parallel imaging reconstruction. Specialized coils that can be integrated with the transducer are in early stages of development, and may offer modest parallel imaging acceleration [17–19]. However, integrated coils are not yet widely available, and may still benefit from combination with other accelerated imaging approaches such as the one described here.

Multiple groups have developed accelerated temperature mapping methods from undersampled k-space data that exploit temporal correlations between and among baseline (pre-treatment) and dynamic (during treatment) images to suppress undersampling artifacts [20–22]. However, adaptations of these methods for brain applications [10, 11] could be affected by signal variations that are not accounted for in signal models, and are not captured in baseline images due to their random dynamic behavior (Fig. 1 b). This breaks temporal correlation assumptions between images collected during a single focused ultrasound sonication, and results in temperature map artifacts.

We present a spatially-segmented approach for reconstructing temperature maps in brain MRgFUS, in which we separately estimate a water bath image without a baseline, and a temperature map in the brain using the k-space hybrid method with a baseline (Fig. 1 c). We compare the approach with temperature maps calculated by the conventional whole-image k-space hybrid method and (when multiple receive coils were used) by phase difference after SENSE image reconstruction [12, 22]. Gel phantom heating data are evaluated using Cartesian and non-Cartesian k-space sampling. We also investigate the effect of reducing the water bath signal intensity on the temperature reconstruction performed with and without spatial segmentation.

## Theory

### Signal model

The spatially-segmented thermometry algorithm reconstructs both a brain temperature map and a water bath image. Inside the brain, the k-space hybrid model is applied which incorporates baseline image data, while no image model is applied in the bath. Given a set of in-brain image voxels $\mathbb {B}$, the signal is modeled as:

1 $$  y_{i} = \sum_{j\in \mathbb{B}} f_{j} e^{-(\alpha_{j}+\imath\theta_{j})} e^{-\imath \vec{k}_{i} \cdot \vec{x}_{j}} + \sum_{j\notin \mathbb{B}} \tilde{f}_{j} e^{-\imath \vec{k}_{i} \cdot \vec{x}_{j}} + \varepsilon_{i},  $$

where *y*_*i*_ is a complex-valued k-space data sample acquired at location $\vec {k}_{i}$, the $\vec {x}_{j}=(x_{j},y_{j},z_{j})$ are spatial locations, the $f_{j} \triangleq f(\vec {x}_{j})$ are samples of the phase drift-compensated in-brain baseline image, the $\tilde {f}_{j} \triangleq \tilde {f}(\vec {x}_{j})$ are samples of the current bath image, the $\alpha _{j} \triangleq \alpha (\vec {x}_{j})$ are samples of a heat-induced image magnitude attenuation map, the $\theta _{j} \triangleq \theta (\vec {x}_{j})$ are samples of the heat-induced phase shift map, $\imath = \sqrt {-1}$, and the *ε*_*i*_ are i.i.d. complex Gaussian noise samples [22, 23]. Here, *θ*_*j*_=*α*_*j*_=0 is assumed for $j\notin \mathbb {B}$.

The hybrid referenceless and multibaseline thermometry model [24] is enforced in the brain, as:

2 $$  f_{j} = \left(\sum_{l=1}^{N_{b}} b_{l,j}w_{l} \right) e^{\imath \left\{\textit{\textbf{Ac}}\right\}_{j}},  $$

where *N*_*b*_ is the number of complex baseline brain images ***b*** reconstructed from fully-sampled k-space data acquired prior to treatment, the *w*_*l*_ are baseline image weights, ***A*** is a matrix of polynomial basis functions, and ***c*** is a vector of polynomial coefficients. Using a weighted combination of baseline images allows robust thermometry over a range of tissue positions and has previously been shown to benefit brain thermometry for MRgFUS [25], though a single baseline will be used in the present work. Background phase drift is modeled by the low-order polynomial basis functions in the matrix ***A***, to account for spatially-smooth dynamic changes in the magnetic field, such as result from *B*_0_ field drift and respiration. Incorporating this model for the baseline image, Eq. 1 can be written as:

3 $$ \begin{aligned}  y_{i} &= \sum_{j\in \mathbb{B}} \left(\sum_{l=1}^{N_{b}} b_{l,j}w_{l}\right) e^{\imath\left\{\textit{\textbf{Ac}}\right\}_{j}} e^{-\left(\alpha_{j}+\imath\theta_{j} \right)} e^{-\imath \vec{k}_{i} \cdot \vec{x}_{j}}\\ &\quad + \sum_{j\notin \mathbb{B}} \tilde{f}_{j} e^{-\imath \vec{k}_{i} \cdot \vec{x}_{j}} + \varepsilon_{i}. \end{aligned}  $$

Figure 1 c illustrates the overall undersampled dynamic image model. Brain voxels are defined using a user-defined region of interest (ROI) mask. As the patient is immobilized in the scanner, preventing translational motion during treatment, the ROI can be defined once for each treatment session.

### Problem formulation

The signal model (Eq. 3) is fit to acquired k-space data contained in a vector ${\tilde {\textit {\textbf {y}}}}$ by solving the following optimization problem:

4 $$ \begin{array}{ll} \text{minimize} & \frac{1}{2} \left\Vert \tilde{\textit{\textbf{y}}} - \textit{\textbf{y}}(\textit{\textbf{w}},\textit{\textbf{c}},\boldsymbol{\alpha},\boldsymbol{\theta},\boldsymbol{\tilde{f}}) \right\Vert_{2}^{2} + \lambda \Vert \boldsymbol{{\alpha}} \Vert_{1} + \lambda \Vert \boldsymbol{{\theta}} \Vert_{1} \\&~~~+ \beta R(\boldsymbol{{\alpha}}+\imath\boldsymbol{{\theta}}) + \eta R(\boldsymbol{{\tilde{f}}}),\\ \textrm{subject to} & \boldsymbol{{\alpha}} \geq 0 \\ & \boldsymbol{{\theta}} \leq 0 \\ & \sum_{l=1}^{N_{b}} w_{l} = 1 \\ & \textit{\textbf{w}} \geq 0, \end{array}   $$

where ∥***·***∥_1_ is the *ℓ*_1_ norm, *λ* is an *ℓ*_1_ regularization parameter that controls the sparsity of ***α*** and ***θ***, and *R*(*·*) is a second-order finite differencing spatial roughness penalty with regularization parameters *β* and *η* that control the smoothness of ***α***, ***θ***, and $\tilde {\textit {\textbf {f}}}$ [22, 26]. This problem is solved by alternately updating the water bath image, and the brain image model parameters, as described next.

### Algorithm

The following alternating minimization algorithm is used to solve the problem in Eq. 4, given initial estimates of ***w***,***c***,***α***,***θ***, and $\boldsymbol {{\tilde {f}}}$: 1: **repeat** 2: Update the water bath image $\boldsymbol {{\tilde {f}}}$, by solving:

5 $$ \begin{array}{ll} \text{minimize} & \frac{1}{2} \sum_{i = 1}^{N_{k}} \left\vert \tilde{y}_{i}^{\neg\mathbb{B}} - \sum_{j\notin \mathbb{B}} \tilde{f}_{j} e^{-\imath \vec{k}_{i} \cdot \vec{x}_{j}} \right\vert^{2} + \eta R(\boldsymbol{{\tilde{f}}}), \end{array}   $$

where *N*_*k*_ is the total number of k-space samples, and $\tilde {\textit {\textbf {y}}}^{\neg \mathbb {B}}$ is the residual k-space signal due to the water bath:

6 $$ \tilde{y}^{\neg\mathbb{B}}_{i} \triangleq \tilde{y}_{i} - \sum_{j\in \mathbb{B}} f_{j} e^{-(\alpha_{j}+\imath\theta_{j})} e^{-\imath \vec{k}_{i} \cdot \vec{x}_{j}}.   $$

This is solved using a conjugate gradient (CG) (single receive coil) or CG-SENSE (multiple receive coils) algorithm [26–28]. Upon updating $\tilde {\textit {\textbf {f}}}$, the residual k-space signal $\tilde {\textit {\textbf {y}}}^{\mathbb {B}}$ due to the brain is updated:

7 $$ \tilde{y}^{\mathbb{B}}_{i} \triangleq \tilde{y}_{i} - \sum_{j\notin \mathbb{B}} \tilde{f}_{j} e^{\imath \vec{k}_{i} \cdot \vec{x}_{j}},   $$

and used in the subsequent brain model parameter updates. 3: Update ***w*** by solving the quadratic programming problem:

8 $$ \begin{array}{ll} \text{minimize} & \frac{1}{2} \left\Vert \tilde{\textit{\textbf{y}}}^{\mathbb{B}} - \textit{\textbf{G}} \text{diag}\left\{ e^{\imath\left(\left\{\textit{\textbf{Ac}}\right\}_{j}\right)} e^{-\left(\alpha_{j}+\imath\theta_{j} \right)} \right\} \textit{\textbf{B}}\textit{\textbf{w}} \right\Vert^{2} \\ \textrm{subject to} & \sum_{l=1}^{N_{b}} w_{l} = 1 \\ & \textit{\textbf{w}} \geq 0 \\ & j\in \mathbb{B}, \end{array}   $$

where ***G*** is a discrete Fourier Transform (FT) matrix and ***B*** is a matrix whose columns are the baseline images ***b*** [22]. 4: Update ***α*** and ***θ*** by solving the constrained minimization problem:

9 $$ \begin{array}{ll} \text{minimize} & \frac{1}{2} \sum_{i=1}^{N_{k}} \left\vert \tilde{y}_{i}^{\mathbb{B}} - \sum_{j\in \mathbb{B}} f_{j} e^{-(\alpha_{j}+\imath\theta_{j})} e^{-\imath \vec{k}_{i} \cdot \vec{x}_{j}} \right\vert^{2}\\ &~~+ \lambda \sum_{j} \vert \alpha_{j} \vert + \vert \theta_{j} \vert \\ \textrm{subject to} & \alpha_{j} \geq 0, \\& \theta_{j} \leq 0, \end{array}   $$

using a nonlinear conjugate gradient (NLCG) algorithm as described in Ref. [22]. 5: Update ***c*** using an NLCG algorithm that is similar to the ***α*** and ***θ*** updates, but incorporates the basis matrix ***A*** and applies no sparsity regularization or sign constraints. This is further described in Ref. [22]. 6: **until** Stopping criterion met. 7: To eliminate temperature bias due to the *ℓ*_1_ norm, steps 1–6 are repeated with *λ*=0, and ***α*** and ***θ*** are only updated in voxel locations *j* in which *θ*_*j*_ is more negative than a threshold value after Step 6.

## Methods

### Algorithm implementation

All reconstructions and evaluations were performed in MATLAB R2015a (Mathworks, Natick, MA) on a workstation with dual 6-core 2.8 GHz X5660 Intel Xeon CPUs (Intel Corporation, Santa Clara, CA) and 96 GB of RAM. Nonuniform fast Fourier transforms were used for reconstructions from non-Cartesian k-space trajectories [28]. No parallelization was used beyond intrinsically multithreaded MATLAB functions.

Initial values for ***c***,***α***,***θ***, and $\boldsymbol {\tilde {f}}$ were set to zero. The initial baseline image weights, ***w***, were then determined according to Eqs. 7 and 8. The algorithm stopping criterion was a relative change in the objective function of less than 0.1% between consecutive iterations. Estimates of image magnitude attenuation and temperature shift were corrected for bias due to the *ℓ*_1_ norm in voxels where ***θ*** was more negative than −0.01 radians, as described in step 7 of the algorithm. The backtracking line search used in the NLCG algorithm to update ***α*** and ***θ***, described in Ref [22], exited when the relative change in the objective function was less than 0.1% and 0.001% between consecutive iterations for phantom and in vivo datasets, respectively.

### Experimental data

#### Phantom heating experiment

##### Imaging

A gel-filled human skull phantom was sonicated by an Insightec ExAblate Neuro 4000 transcranial MRgFUS system (Insightec Ltd, Haifa, Israel) and imaged at 3T using the body coil (MR750, GE Healthcare, Waukeshaw, WI) [29]. 2DFT gradient echo images were collected with 13 ms TE, 28 ms TR, 28 ×28×0.3 cm ^3^ field of view, 256 ×128 acquisition matrix, and 30° flip angle. Images and maps were reconstructed to a 128 ×128 matrix.

##### Effect of water bath signal level

The signal intensity of the image in the water bath was manually scaled to 0, 25, 50, 75, and 100% of its original value, prior to synthesizing the sampled k-space data, to evaluate the effect of its presence in whole-image and spatially-segmented reconstruction approaches. Temperature reconstructions were performed for 3 × undersampled 2DFT data as described below.

##### Undersampled temperature reconstruction

**Fig. 2:**
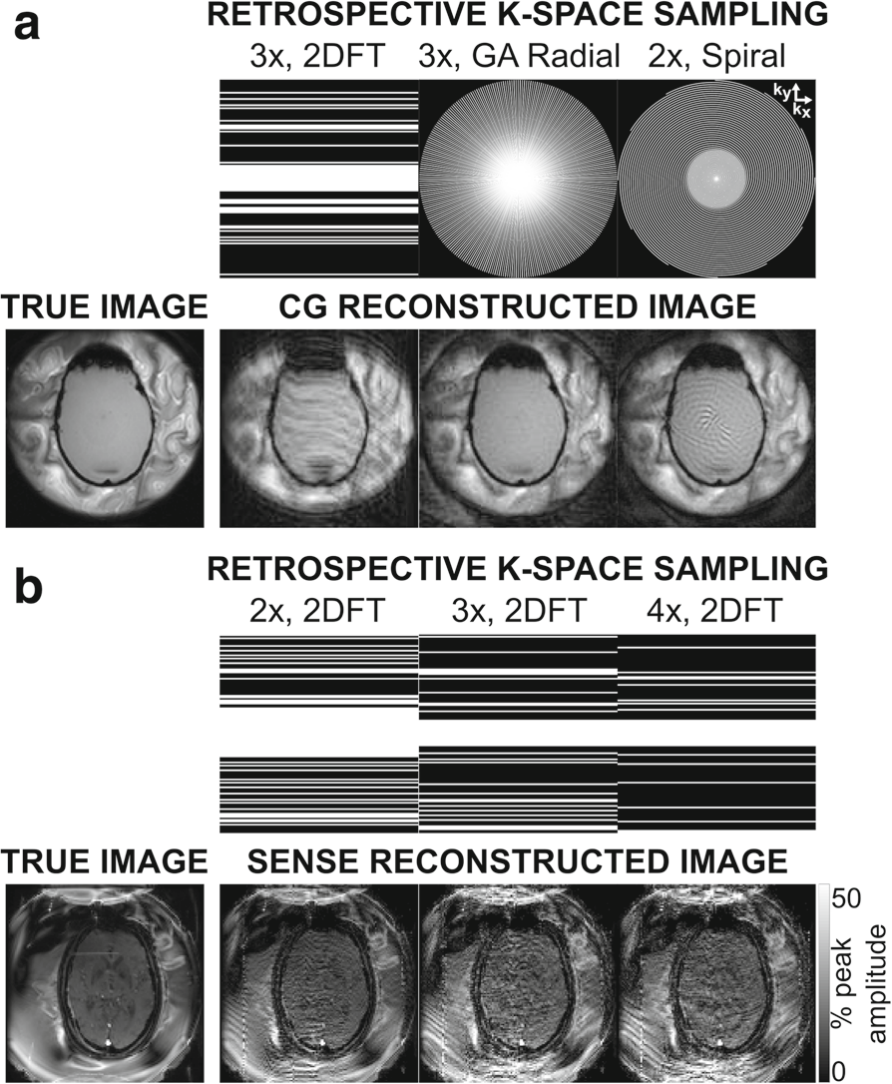
*Retrospective k-space sampling* patterns and *reconstructed magnitude images*. **a** Gel phantom heating. *k-Space sampling* patterns for 3 ×-accelerated 2DFT and *golden angle radial*, and 2 ×-accelerated *spiral trajectories*. The original image and images reconstructed from each undersampled trajectory are *shown below*. **b** In vivo MRgFUS treatment. *k-Space sampling* patterns for 2, 3, and 4 ×-accelerated 2DFT trajectories. The original image and images reconstructed from each undersampled trajectory are *shown below* (intensity level windowed to show image detail)

2DFT data from the phantom heating experiment were retrospectively undersampled by 1, 2, 3, and 4 × (128, 64, 42, and 32 total lines), with full sampling over 24 (2 ×) and 17 (3 and 4 ×) central k-space lines. Fully-sampled k-space data were also resampled onto golden angle (GA) radial and variable density spiral trajectories using a non-uniform fast Fourier transform [28]. GA radial data were undersampled by 1, 2, 3, and 4 × (202, 101, 67, and 50 lines). Spiral data were undersampled by 1, 1.5, 2, 2.4, and 3 × (24, 16, 12, 10, and 8 interleaves) with full sampling over the central 25−30% of k-space). Figure 2 a illustrates k-space sampling patterns and CG image reconstructions of the undersampled data for each trajectory.

Temperature change maps of phantom heating were reconstructed using the conventional whole-image k-space hybrid method (“k-space everywhere”), and the proposed method (“spatially-segmented”). Regularization parameters for the k-space hybrid temperature model and the number of iterations *n* used in the CG reconstruction were: *λ* = 10^−5^, *β* = 10^−5.25^, and *n*=2 for 2DFT; *λ* = 10^−10^, *β* = 10^−4.4^, and *n*=4 for GA radial; and *λ* = 10^−20^, *β* = 10^−20^, and *n*=16 for spiral. The ROI corresponding to the gel phantom brain was selected from a fully-sampled image. An 8 ×8-voxel square ROI centered on the temperature hot spot was defined to calculate mean and peak temperature changes and temperature error. Root-mean-square error (RMSE) was also computed between the reconstructed and fully-sampled images in the brain and hot spot ROIs.

#### MRgFUS thalamotomy

##### Imaging

A patient received MRgFUS thermal ablation treatment at 3T (Signa Excite, GE Healthcare, Milwaukee, WI; ExAblate Neuro, Insightec Ltd., Haifa, Israel) as part of a chronic neuropathic pain treatment study at the University Hospital of Zurich. Full informed written consent was obtained prior to the treatment. 2DFT gradient echo images were collected with an 8-channel receive coil that wrapped around the outside of the transducer (RAPID Biomedical, Rimpar, Germany), a 13 ms TE, 28 ms TR, 28 ×28×0.3 cm ^3^ field of view, 256 ×128 acquisition matrix, and 30° flip angle.

##### Undersampled temperature reconstruction

Images and maps were reconstructed to a 128 ×128 matrix and retrospectively undersampled by 2, 3, and 4 × (64, 42, and 32 lines), with full sampling over 32 (2 ×) and 17 (3 and 4 ×) central k-space lines. SENSE coil sensitivity maps were estimated by reconstructing the average k-space data across dynamics and dividing by the sum-of-squares image [12]. Figure 2 b shows k-space sampling and SENSE image reconstructions of the undersampled data for each acceleration factor.

Temperature maps were calculated by phase difference between baseline and dynamic SENSE-reconstructed images (“SENSE everywhere”), and using the segmented method with CG-SENSE reconstruction of the water bath image (“spatially-segmented”). Temperature maps derived from the SENSE-reconstructed images incorporated the background phase drift correction estimated by the spatially-segmented k-space brain model. k-Space hybrid regularization parameters and iterations per CG-SENSE bath image update were: *λ* = 10^−6.265^, *β* = 10^−20^, and *n*=2. An ROI mask of the brain mask was selected from a fully-sampled baseline image. A 4 ×4-voxel square ROI centered on the temperature hot spot was defined to calculate mean and peak temperature changes. RMSE was calculated as for the phantom data.

## Results

### Phantom heating

**Fig. 3:**
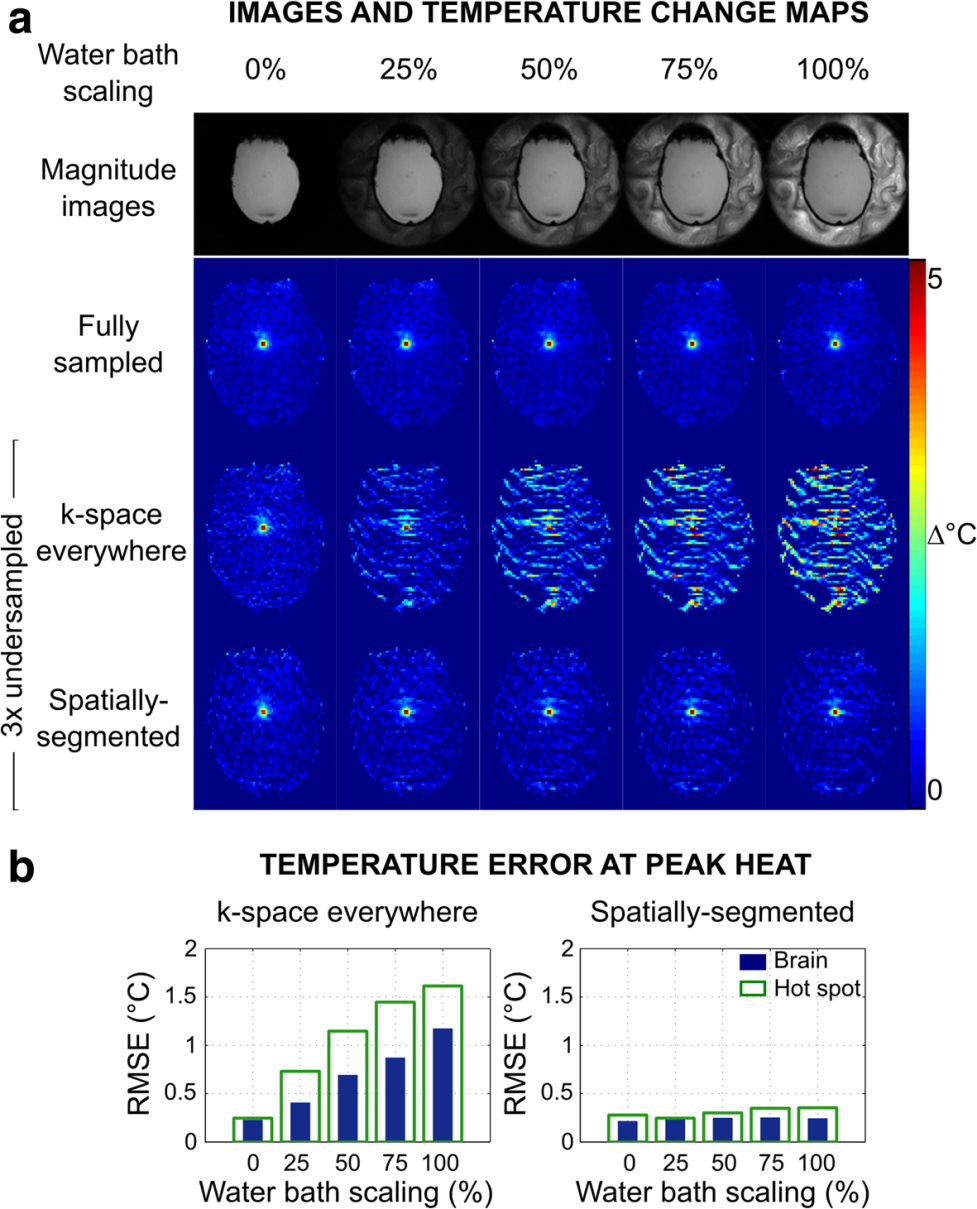
Water bath scaling results. **a** *Magnitude images* and *temperature maps* reconstructed at *peak heat* with full 2DFT sampling and 3 × undersampling, with image intensity in the *water bath* scaled from 0-100% in baseline and dynamic images, prior to synthesizing the undersampled k-space data. **b** Root-mean-square error versus water bath signal scaling for undersampled temperature reconstructions

**Effect of water bath signal level** Figure 3 shows temperature maps and errors at peak heat for images in which the water bath was scaled between 0 and 100% of its true value. With full data sampling, the water bath signal level does not affect the temperature map error. However, as the water bath signal increases to its true value, errors arise in temperature maps estimated from undersampled data without spatial segmentation. With zero signal in the water bath, temperature estimates from k-space everywhere and spatially-segmented methods are similar. Temperature maps reconstructed using the spatially-segmented algorithm are also similar in appearance and RMSE across the range of water bath image scaling levels. The RMSE in the brain/hot spot was improved by factors of 1.06/0.89 (0% scaling), 1.75/2.97 (25% scaling), 2.81/3.81 (50% scaling), 3.47/4.16 (75% scaling), and 4.85/4.56 (100% scaling) using the spatially-segmented versus k-space everywhere reconstruction.

**Fig. 4:**
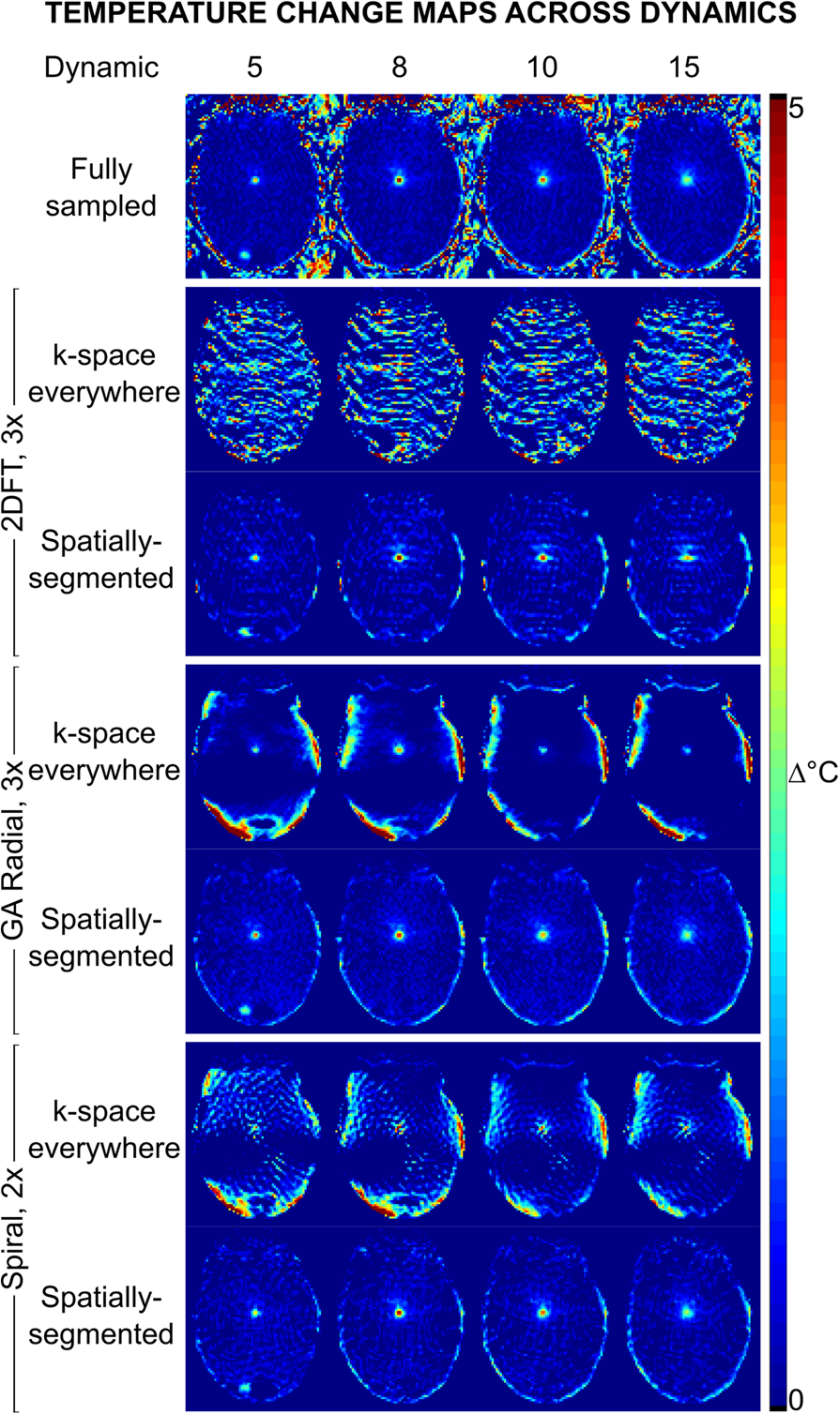
Phantom heating results. Reconstructed temperature changes in the brain phantom with 3 × undersampled 2DFT and GA radial, and 2 × undersampled spiral trajectories

**Undersampled temperature reconstructions** Temperature maps reconstructed from undersampled data during heating (dynamic 5), peak heat (dynamic 8), and cooling (dynamics 10 and 15) are displayed in Fig. 4. Across all dynamics of the 3 × undersampled 2DFT data, temperature maps have high error when reconstructed using the k-space everywhere method. With GA radial sampling, 3 × undersampled k-space everywhere reconstructions have much lower in-brain artifact, although errors are observed near the periphery of the brain. Compared to 2DFT, 2 × undersampled spiral k-space everywhere reconstructions have lower in-brain artifact, though errors are present throughout the brain that are similar in appearance to the CG reconstruction errors in the magnitude image (Fig. 2 a). All spatially-segmented reconstructions have low temperature error in the brain.

**Fig. 5:**
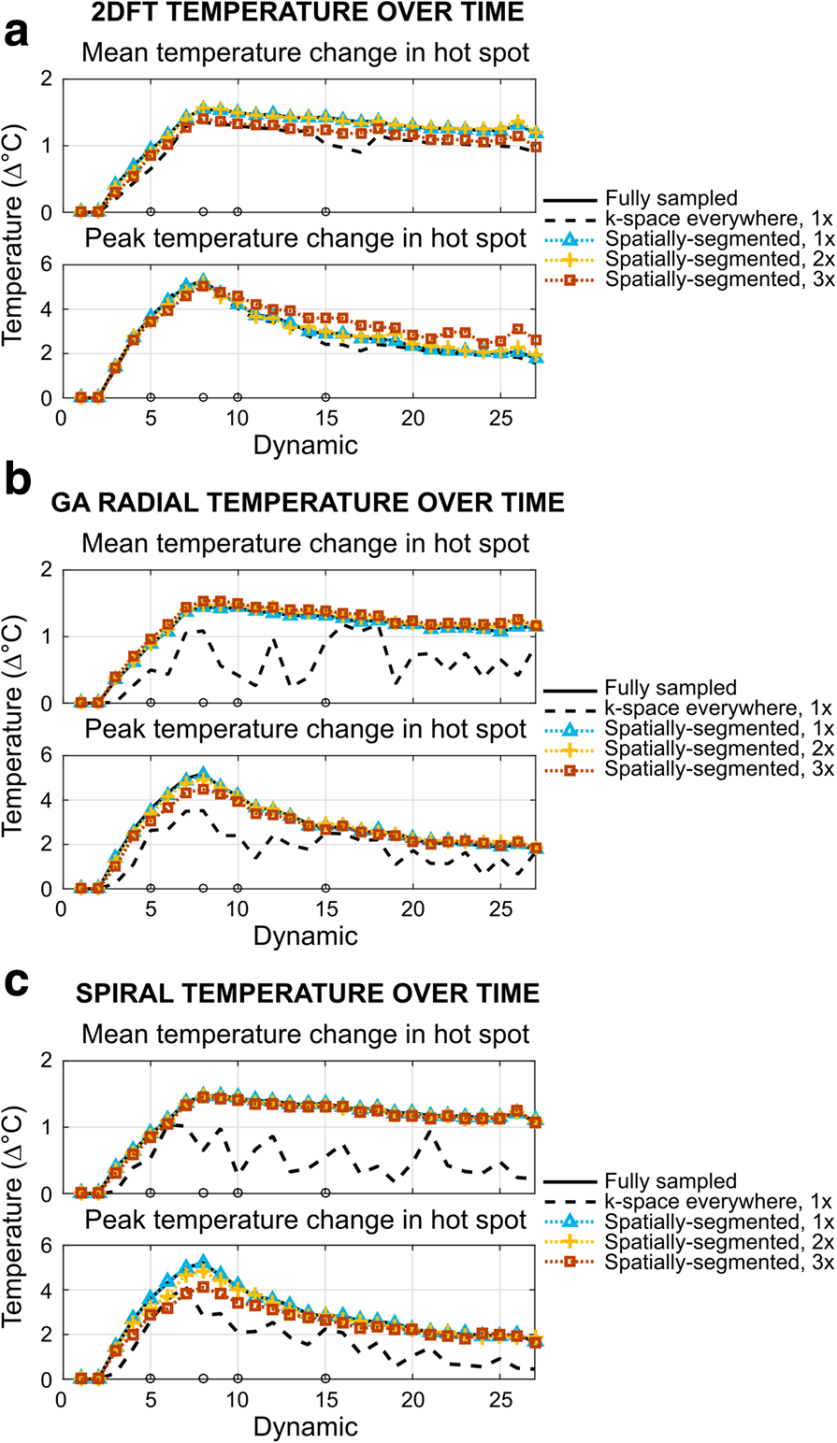
Mean and peak temperature changes in the brain phantom. Mean and peak reconstructed temperature change in the hot spot for (**a**) 2DFT, (**b**) golden angle radial, and (**c**) spiral trajectories (circles on x-axis indicate dynamics of displayed maps in Fig. 4)

Figure 5 shows mean and peak temperature change in the hot spot for fully sampled and undersampled reconstructions for each trajectory. Mean and peak temperature change estimates contain errors using the k-space everywhere reconstruction, even with no undersampling, since unaccounted for signal differences in the water bath between baseline and dynamic images create errors in fitting the temperature change model to the data. As acceleration increases, 2DFT estimates of peak temperature change are slightly overestimated during cool-down dynamics, and GA radial and spiral estimates of peak heat are slightly dampened using the proposed method. In all cases, spatially-segmented reconstructions tracked the average temperature change in the hot spot within 0.24 °C. Spatially-segmented 2DFT reconstructions tracked the peak temperature rise within 0.89 °C at all factors; GA radial and spiral reconstructions tracked within 0.94 °C for factors up to 4 × (GA radial) and 2.4 × (spiral).

**Fig. 6:**
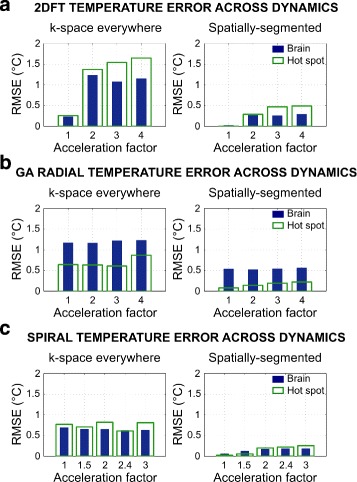
RMSE of reconstructed brain phantom temperature maps. Root-mean-square error in the phantom brain and temperature hot spot for (**a**) 2DFT, (**b**) golden angle radial, and (**c**) spiral trajectories across acceleration factors

RMSE is lower for spatially-segmented reconstructions compared to k-space everywhere across all image dynamics in both the brain and hot spot (Fig. 6). On average, RMSE in the brain/hot spot was reduced by factors of: 5.96/26.77 (2DFT), 2.20/4.91 (GA radial), and 5.65/12.00 (spiral).

### MRgFUS thalamotomy

**Fig. 7:**
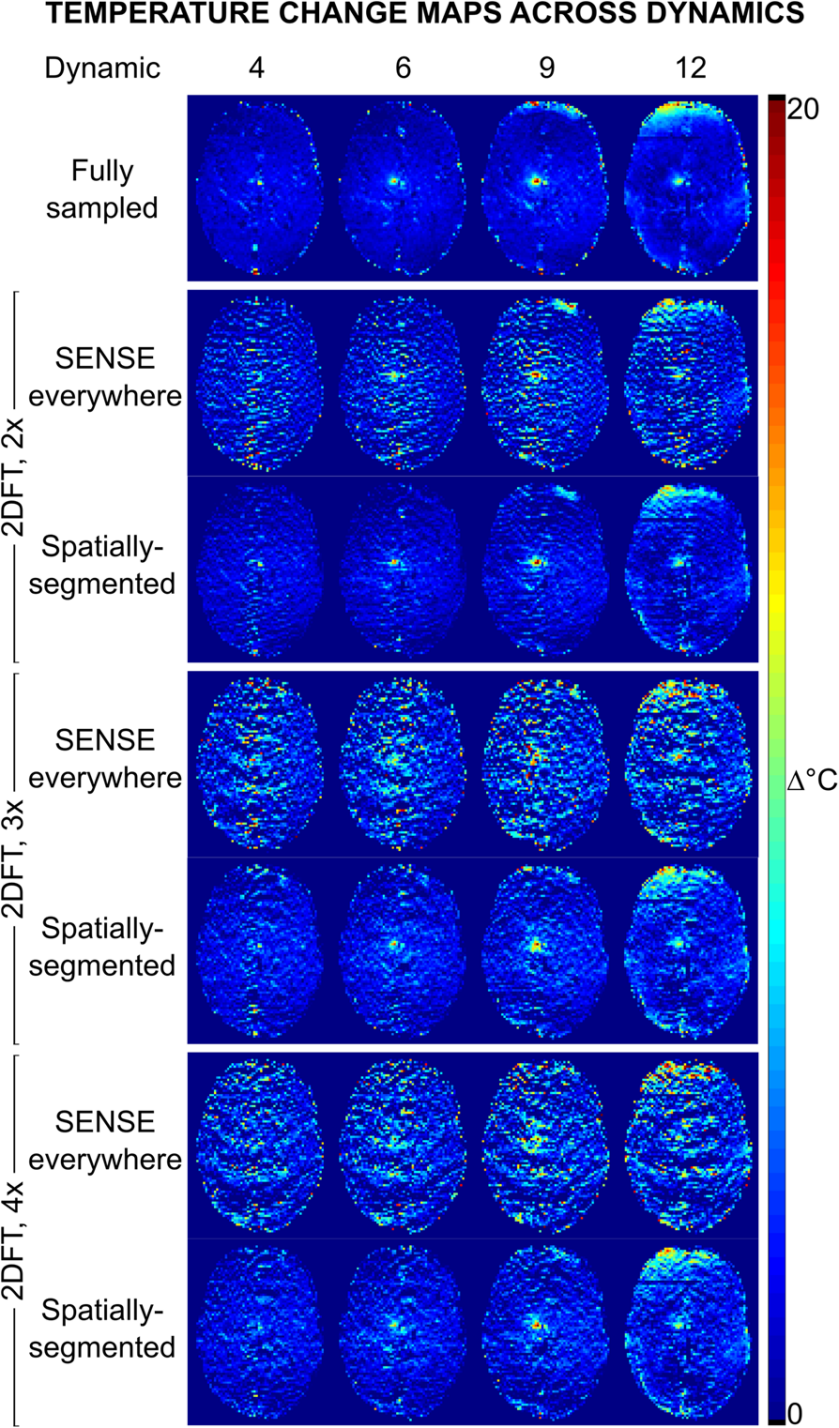
In vivo MRgFUS treatment results. Reconstructed temperature change maps in the brain across dynamics from fully sampled and 2-4 × undersampled 2DFT data

**Fig. 8:**
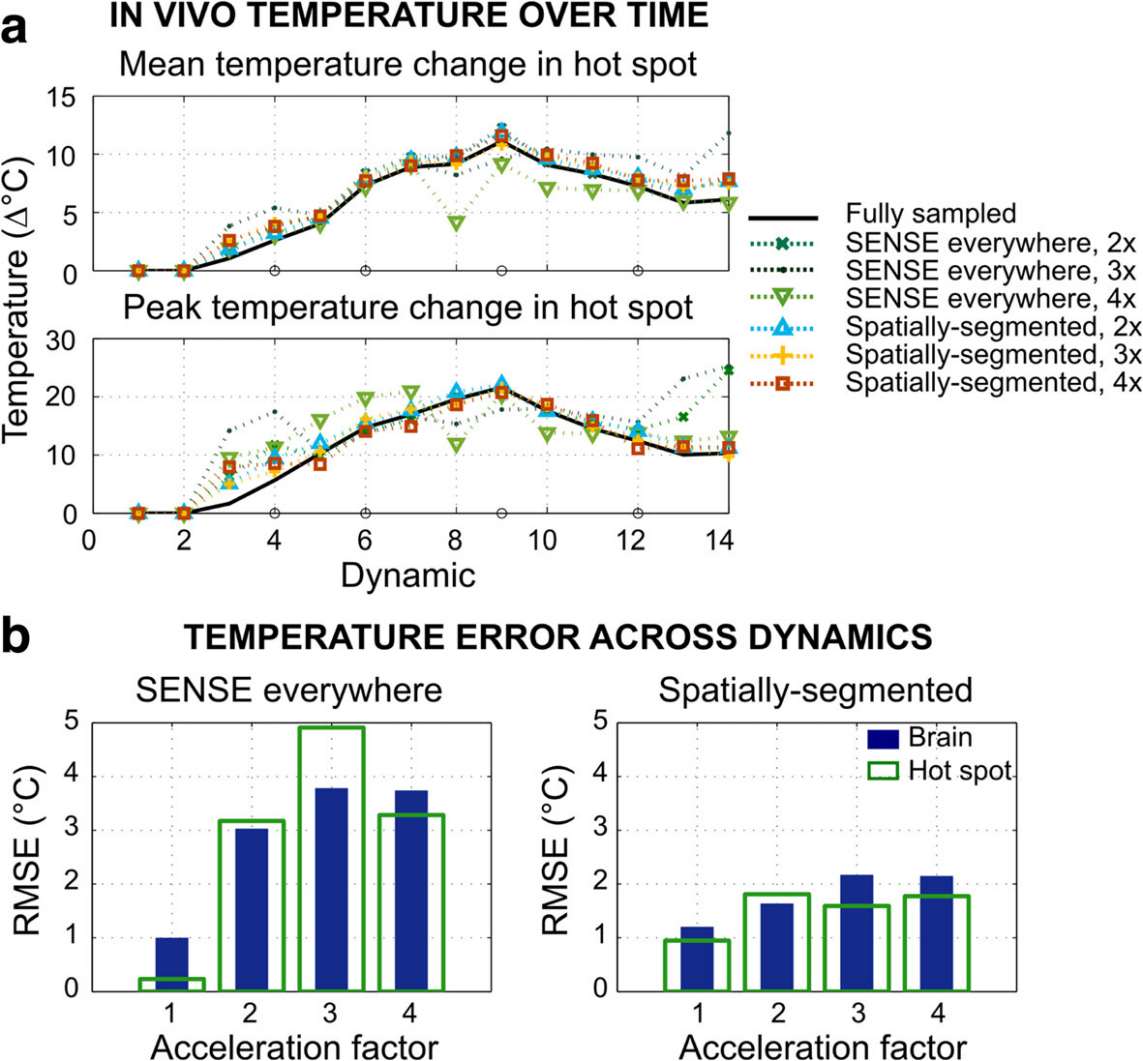
In vivo MRgFUS treatment results. **a** Mean and peak temperature change in the hot spot is *plotted* for each reconstruction (*circles* on x-axis indicate dynamics of displayed maps in Fig. 7) (**b**) Root-mean-square error in the brain and hot spot for accelerated temperature reconstructions

Figures 7–8 show reconstruction results from the in vivo MRgFUS thermal ablation treatment. At 2 ×, temperature estimates from SENSE reconstructed images are similar to fully sampled maps in the hot spot, but contain large errors within the brain. SENSE-reconstructed images contain significant aliasing artifacts (Fig. 2 b) that degrade temperature map accuracy in the brain. At 3 × and 4 ×, increased phase artifacts obscure the hot spot and cause higher temperature error across image dynamics. Artifacts are lower in all the spatially-segmented temperature maps. At all factors, the spatially-segmented reconstruction tracked the average temperature change within 1.53 °C, and tracked the peak temperature rise within 3.38 °C up to 3 ×, reflecting slightly higher temperature error at dynamic 3. Excluding dynamic 3, the peak temperature estimate was within 1.52 °C up to 3 ×. RMSE in the brain/hot spot is reduced using the proposed method compared to SENSE by factors of 1.85/1.75 (2 ×), 1.75/3.09 (3 ×), 1.74/1.85 (4 ×). The total number of iterations and compute time to reconstruct the temperature map at the time frame corresponding to the peak of heating was: 42 iterations and 37 s (2 ×); 86 iterations and 76 s (3 ×); and 136 iterations and 110 s (4 ×), without parallelization or other optimizations for speed.

## Discussion

### Summary of main results

Unpredictable water bath motion during brain MRgFUS confounds model-based approaches to accelerated MR temperature mapping, resulting in large temperature artifacts due to aliasing of water bath signals into the brain. The proposed spatially-segmented reconstruction approach was demonstrated to reduce error in undersampled temperature reconstructions of a gel-filled human skull ablation with a single receive coil, which is the most common coil configuration currently, and an in vivo thermal ablation with 8 receive coils, which may become the most common coil configuration in the near future. Rather than relying on previously acquired baseline images, separately reconstructing the image in the water bath at each treatment dynamic better characterizes its signal and results in lower temperature error in the brain.

Phantom heating experiments demonstrated errors in undersampled temperature reconstruction when using the baseline water bath image as a reference for the treatment image, even when water bath signal intensity was reduced to 25% of its actual value. However, reconstructing the water bath image at each dynamic and applying a model-based temperature reconstruction in the brain resulted in undersampled temperature maps with low error using a single receive coil with 2DFT, golden angle radial, and variable density spiral k-space sampling trajectories, regardless of the water signal strength. This indicates that the proposed method could be of value, even if water is doped to reduce its signal.

In vivo data demonstrated the spatially-segmented reconstruction approach achieved low temperature error compared with temperature maps calculated from images reconstructed with SENSE. Magnitude images estimated by the k-space hybrid method in the brain (derived from the input baseline images and corrected for phase drifts) and CG in the water bath also had lower error than SENSE reconstructions of the dynamic images (results not shown). Overall, the segmented method is complementary to parallel reception, since parallel imaging reconstruction will perform better in the water bath where the multiple coils have more distinct sensitivities, but less well in the brain where the sensitivities are similar and do not provide distinct encoding. By using prior baseline information in the temperature model, the segmented method achieves good reconstructions in the brain.

### Reconstruction of the image in the water bath

Early attempts to incorporate compressed sensing using standard wavelet *ℓ*_1_ penalties did not significantly improve temperature reconstruction results. However, it is possible that using better sparsifying transforms that are tailored to the water bath could enable the use of a compressed sensing reconstruction in the water bath. Improved water bath image reconstruction could potentially reduce computation time, by reducing the number of iterations required in the reconstruction.

### Modifications to reduce MR signal intensity in the water bath

A possible solution to reduce temperature error associated with the water bath is to alter the water to have low MR signal intensity. An acceptable contrast agent would need to be both biologically safe and acoustically transparent. Although deuterated water (^2^ *H*_2_O, or D _2_O) has low MR signal, it has been shown to have negative effects on cell function and structure, suggesting that dosage and safety effects would need to be investigated before adopting a D _2_O solution in the bath [30–32].

Gadolinium (Gd) could be added to the water to decrease its T _1_ relaxation time. While Gd is also toxic, chelated forms such as Gd-diethylenetriaminepentacetate (Gd-DTPA) have been used safely in patients. However, high Gd-DTPA concentrations increase the inhomogeneity of the local magnetic field, causing signal loss in nearby pixels [33–35]. While this could be ignored in the water bath itself, it may impair safety monitoring near the skull surface, where the risk of tissue overheating is high. Studies in tissue have shown the Gd-DTPA structure is not disrupted by the application of ultrasound [36]. However, investigation of Gd-DTPA in the water bath may be warranted to determine whether there is any negative impact on the chelate structure, ultrasound wave propagation, or radiofrequency wave conduction.

### Computational considerations

Computation times for the current implementation of the algorithm were on the order of tens of seconds per time frame, which is not compatible with real-time clinical use. Real-time clinical use will require more powerful computing, parallelization, and algorithm innovations to reduce compute time by approximately a factor of 100, so that each time frame’s temperature map is fully computed before data acquisition for the next frame is completed. For example, a finely parallelized GPU implementation could dramatically accelerate and even obviate the use of non-uniform fast Fourier transforms for non-Cartesian reconstructions [37]. However, the method could immediately be used for pre-clinical applications, as well as in-between clinical sonications to obtain the best possible temperature maps retrospectively for treatment verification and guidance.

### Other possible embodiments

Although the method presented here was demonstrated with the k-space hybrid dynamic image model, it should be compatible with other accelerated temperature mapping methods [20]. The segmented approach may also be useful to suppress temperature artifacts due to intra-scan water bath motion in fully-sampled acquisitions. Finally, it may find applications outside the brain in scenarios where the sonicated target region does not move, but there is other organ motion distant from the target (such as bowel motion in uterine fibroid treatments).

## Conclusions

While the water bath enables transcranial applications of MRgFUS by providing acoustic coupling and cooling, it presents unique challenges in the reconstruction of temperature maps, particularly from undersampled MRI data. Applying separate reconstructions to the image in the brain and water bath results in lower temperature error when undersampling k-space using single and multiple receive coils. The spatially-segmented reconstruction method enables temperature estimation with low artifacts from undersampled data during brain MRgFUS treatments, and can be combined with parallel imaging methods when multiple receive coils are available.

## Abbreviations

2DFT: Two-dimensional Fourier transform
CG: Conjugate gradient
GA: Golden angle
RMSE: Root-mean-square error
ROI: Region of interest
MRgFUS: Magnetic resonance-guided focused ultrasound
NLCG: Nonlinear conjugate gradient
SENSE: Sensitivity encoding
TE: Echo time
TR: Repetition time
